# Design and Biophysical Characterization of Poly (l-Lactic) Acid Microcarriers with and without Modification of Chitosan and Nanohydroxyapatite

**DOI:** 10.3390/polym10101061

**Published:** 2018-09-25

**Authors:** Liying Li, Kedong Song, Yongzhi Chen, Yiwei Wang, Fangxin Shi, Yi Nie, Tianqing Liu

**Affiliations:** 1 State Key Laboratory of Fine Chemicals, Dalian R&D Center for Stem Cell and Tissue Engineering, Dalian University of Technology, Dalian 116024, China; liyingli@mail.dlut.edu.cn (L.L.); chenyongzhi@mail.dlut.edu.cn (Y.C.); 2 Burns Research Group, ANZAC Research Institute, Concord, University of Sydney, Sydney, NSW 2139, Australia; eweiwang@hotmail.com; 3 Zhengzhou Institute of Emerging Technology Industries, Zhengzhou 450000, China; Shifangxin1122@163.com; 4 Key Laboratory of Green Process and Engineering, Institute of Process Engineering, Chinese Academy of Sciences, Beijing 100190, China

**Keywords:** microcarriers, Poly (l-lactic) acid, Chitosan, nanohydroxyapatite, osteoblasts

## Abstract

Nowadays, microcarriers are widely utilized in drug delivery, defect filling, and cell culture. Also, many researchers focus on the combination of synthetic and natural polymers and bioactive ceramics to prepare composite biomaterials for tissue engineering and regeneration. In this study, three kinds of microcarriers were prepared based on physical doping and surface modification, named Poly (l-lactic) acid (PLLA), PLLA/nanohydroxyapatite (PLLA/nHA), and PLLA/nHA/Chitosan (PLLA/nHA/Ch). The physicochemical properties of the microcarriers and their functional performances in MC3T3-E1 cell culture were compared. Statistical results showed that the average diameter of PLLA microcarriers was 291.9 ± 30.7 μm, and that of PLLA/nHA and PLLA/nHA/Ch microcarriers decreased to 275.7 ± 30.6 μm and 269.4 ± 26.3 μm, respectively. The surface roughness and protein adsorption of microcarriers were enhanced with the doping of nHA and coating of chitosan. The cell-carrier cultivation stated that the PLLA/nHA microcarriers had the greatest proliferation-promoting effect, while the PLLA/nHA/Ch microcarriers performed the strongest attachment with MC3T3-E1 cells. Besides, the cells on the PLLA/nHA/Ch microcarriers exhibited optimal osteogenic expression. Generally, chitosan was found to improve microcarriers with superior characteristics in cell adhesion and differentiation, and nanohydroxyapatite was beneficial for microcarriers regarding sphericity and cell proliferation. Overall, the modified microcarriers may be considered as a promising tool for bone tissue engineering.

## 1. Introduction

Since the concept of microcarriers was first proposed by Van Wezel in 1967 [1], microcarriers with particle size in a certain micrometer range have received extensive attention and development in drug delivery, defect filling, and three-dimensional cell culture [2,3,4,5,6,7,8]. Compared to conventional monolayer cell culture and three-dimensional static culture with a porous scaffold, utilizing microcarriers can increase the specific surface area and achieve the uptake of cells attached on their curved surface within a short period of time. Also, microcarriers are capable of integrating with bioreactors to create a dynamic environment, and cell delivery and passage can be realized by supplementing fresh microcarriers, thereby avoiding the damage caused by enzymatic digestion [9,10,11,12]. Furthermore, some specific microcarriers can also be used as injectable materials to repair defective tissues in vivo [13,14,15].

The biological materials for the fabrication of these microcarriers are mainly derived from natural macromolecule materials, bioactive ceramics (hydroxyapatite, tricalcium phosphate, and glass/ceramic), and biodegradable synthetic polymers [16,17,18]. For example, Lai et al prepared gelatin microcarriers which were functionalized with oxidized hyaluronic acid and their study suggested that the microcarriers have good compatibility with rabbit corneal cells [19]; Song et al [20] grafted thermosensitive polymers onto the surface of glass microcarriers to improve cell adhesion and preserve osteoblasts and BM-MSCs (Rat Bone Marrow Mesenchymal Stem Cells) biological properties after consecutive passages with the thermal-liftoff method. Levato et al [21] made bone marrow mesenchymal stem cells attach to the surface of poly (l-lactic) acid microcarriers, and created an osteochondral double sphere model using gelatin acrylamide gel bio-ink with the help of 3D printing to promote the bidirectional differentiation of bone and cartilage. Among the synthetic materials used in the preparation of microcarriers, poly (l-lactic) acid has been developed rapidly in tissue engineering because of its good biodegradability and mechanical strength; usually it needs to be modified by some kind of surface treatment such as plasma and laser treatment, etching, grafting, etc. because of its poor hydrophilicity and low cytocompatibility [22,23,24,25,26]. As the main inorganic component of the human skeleton, HA (hydroxyapatite) can be firmly combined with bone tissue in vivo and coexist harmoniously with cells. The organic combination of poly (l-lactic) acid and nHA can lead to outstanding biological performance. On one hand, nHA can improve the osteogenic differentiation of adherent cells; on the other hand, it can partially neutralize the acidic degraded products of poly (l-lactic) acid. Studies have proved that the combination of hydroxyapatite and biopolymer materials can have complementary advantages for a specific application [27,28,29,30,31]. Besides, chitosan, as one of the injectable materials, also has good biocompatibility and biodegradability, while chitosan also has the ability to further strengthen the surface affinity of poly (l-lactic) acid [32,33,34]. For instance, Gao et al. [35] modified the surface of poly (l-lactic) acid microcarriers with chitosan, thus enhancing the adhesion and proliferation of chondrocytes.

Although utilizing microcarriers for suspension cultures can enhance cell proliferation and solve the difficulty that some anchorage-dependent cells cannot grow directly in the bioreactors, there are still some problems in the production of microcarriers, such as poor cellular affinity, high cost, and complicated preparation process. Therefore, innovative exploration and development are still needed to manufacture cost-effective and biocompatible microcarriers for practical application. In this study, PLLA, PLLA/nHA, and PLLA/nHA/Ch microcarriers were prepared based on physical doping and surface modification using gelatin as the dispersant. The differences of physicochemical properties of the three kinds of microcarriers and their functional performance in osteoblasts-like cell culture were compared. Through the improvement of PLLA microcarriers to enhance its biological function, this study may provide some reference value for the development of bone tissue engineering.

## 2. Materials and Methods

### 2.1. Materials

Murine MC3T3-E1 Subclone14 cells were purchased from the Cell bank of the Chinese Academy of Sciences. Poly (l-lactic) acid (PLLA, MW 100,000 Da) was purchased from Jinan Daigang Biomaterial Co., Ltd. (Jinan, China) Silver nitrate, gelatin (analytical agent, the purity was greater than 99.5%), chitosan (the degree of deacetylation was around 95%), and nanohydroxyapatite (nHA) were purchased from Beijing Coolaber Science & Technology Co., Ltd. (Beijing, China) 1-(3-dimethylaminopropyl)-3-ethylcarbodiimide hydrochloride (EDC), morpholine ethane sulfonic acid (MES), and *N*-hydroxy-succinamide (NHS) were obtained from Shanghai Macklin Biochemical Technology Co., Ltd. (Shanghai, China) Cell Counting Kit-8 (CCK-8) was purchased from Dojindo Laboratories (Kumamoto, Japan). Calcein-AM, Hoechst 33258, and Propidium Iodide (PI) were purchased from Sigma-Aldrich Inc. (St. Louis, MO, USA). Alkaline phosphatase assay kit, BCA (Bicinchoninic Acid) protein assay kit, and Alizarin Red S reagent were purchased from Nanjing Jiancheng Bioengineering Institute (Nanjing, China). All reagents and solvents were at analytical grade. MC3T3-E1 Cells were cultured in α-Dulbecco’s Modified Eagle’s Medium (α-DMEM, Hyclone, Logan, UT, USA) containing 10% fetal bovine serum (FBS, Minhai, China), 100 units/mL penicillin and 100 μg/mL streptomycin (Hyclone, Logan, UT, USA) in a humidified 5% CO_2_ atmosphere at 37 °C.

### 2.2. Preparation and Characterization of PLLA, PLLA/nHA and PLLA/nHA/Ch Microcarriers

Poly (l-lactic) acid was fully dissolved in dichloromethane at a ratio of 1:10 (*W*/*V*) prior to being added into gelatin aqueous solution which was more biocompatible than other polymer dispersants. Then, the gelatin-poly (l-lactic) acid suspension was stirred for 5 h. The liquid beads gradually solidified and precipitated at the bottom of the beaker with the volatilization of organic solvent. Finally, the wet microcarriers were collected and dried in a vacuum drying oven.

A similar protocol was used to prepare PLLA/nHA microcarriers. A certain amount of nHA was added to the polymer solution, followed by mechanical agitation for two hours with a lid on to reduce the volatilization of dichloromethane. The following steps were the same as above.

Preparation of PLLA/nHA/Ch microcarriers: The PLLA/nHA microcarriers were firstly soaked in 1 M sodium hydroxide solution, and then repeatedly rinsed with 0.1 M dilute hydrochloric acid solutions and water. The cleavage of ester bonds and exposure of carboxyl groups of PLLA can be induced by alkaline treatment. Next, the microcarriers were transferred to the crosslinking agent solution (EDC/NHS/MES) and soaked for two hours, followed by the addition of 1% chitosan acetic acid solution under 45 °C for 5 h. For this reason, the amide bonds were formed between the carboxyl groups of PLLA and the amino groups of chitosan. Then the microcarriers were soaked in EDC/NHS/MES solution again for 3 h and finally washed with 0.1 M disodium hydrogen phosphate solution and deionized water in triplicate [35].

### 2.3. Analysis of Particle Size Distribution

The effect of stirring speed (150 rpm, 200 rpm, 300 rpm), concentrations of PLLA (5%, 10%, 15%), gelatin (0.25%, 0.5%, 1%), and nHA (W/WPLLA: 0%, 10%, 20%) on the particle size distribution of microcarriers was analyzed. A total of 12 experiments were conducted to select suitable preparation conditions. Three different 40× images of microcarriers in each group were taken by an inverted phase contrast microscope. The diameters of 300 microspheres in each group were measured by Image-Pro Plus software (v7.0, Media Cybernetics, Inc., Bethesda, MD, USA), and the histogram of the particle size distribution of microcarriers in each group was drawn based on statistical calculation.

### 2.4. Morphology Observation and Component Analysis of Microcarriers

The surface profile and topography of microcarriers were observed using an inverted phase contrast microscope and scanning electron microscope (SEM, FEI Company, Hillsboro, OR, USA). Semi-quantitative analysis of specific elements of microcarriers was carried out by EDS energy spectrometry, and the content of nitrogen of microcarriers was analyzed using an elemental analyzer (VarioELIII, Elementar, Langenselbold, Germany). The specific chemical functional groups of microcarriers were detected by Fourier Transform Infrared Spectroscopy (FTIR, Nicolet 6700, Thermo Fisher Scientic Inc., Madison, WI, USA).

### 2.5. Protein Adsorption Determination

The BCA protein assay kit was used to determine the protein adsorption capacity of the three kinds of microcarriers. In brief, a certain volume of BSA (Bovine Serum Albumin) solution with microcarriers was set as an experimental group, while the control group was the same BSA solution without microcarriers. After incubation at 37 °C for 12 h, the supernatant was transferred to an EP (eppendorf) tube, followed by the addition of BCA working liquid. The absorbance of the assay solution at the emission wavelength of 562 nm was detected by an Enzyme-linked immune detector (Thermo scientific, Waltham, MA, USA). Then, the protein concentration was calculated according to the standard protein concentration curve. The protein adsorption capacity of microcarriers was measured by the following calculation method: the difference of protein mass between the control group and experimental group was normalized to the mass of microcarriers.

Three kinds of microcarriers of equal weight whose initial mass was taken as m_1_ were immersed in phosphate buffer (PBS) of equal volume for 6 weeks. These microcarriers were collected periodically, and their dry weights were measured and regarded as m_2_. The calculation formula of degradation rate was: $\frac{m_{1}{-m}_{2}}{m_{2}}\times 100\%$.

### 2.6. MC3T3-E1 Cell Culture and Osteogenic Differentiation

The morphology of MC3T3-EI cells was observed by Hematoxylin-Eosin (HE) staining. One hundred microliters of cells suspension was seeded in a 24-well plate at a cell density of 2 × 10^4^ cells/mL. After incubation for 3 h, the osteogenic medium (α-DMEM, 10%FBS, 50 μg/mL ascorbic acid, and 10 mmol/L beta sodium phosphate) was added. The osteogenic differentiation ability of MC3T3-E1 cells was determined by alkaline phosphatase (after incubation for one week), silver nitrate (Von-Kossa) (three weeks), and Alizarin Red S staining (four weeks).

### 2.7. Inoculation and Culture of MC3T3-E1 Cells on Microcarriers

The microcarriers were soaked in a 75% ethanol solution and simultaneously irradiated with ultraviolet light for 5 h, and finally rinsed with aseptic PBS 3 times. The sterilized microcarriers were reserved for cells culture. The MC3T3-E1 cells at passage 7 were used for the subsequent study. The three kinds of microcarriers of equal quality (50 mg/per well) were respectively placed in a low adhesion 24-well plate, followed by seeding with 50 μL of cell suspension with a density of 2 × 10^6^ cells/mL. The medium was refreshed every 2 days. Then, the cells–microcarriers were stained with Calcein-AM/PI/Hoechst 33258 dye after cultured for 24 h and 120 h, which was observed under a fluorescence microscope. At the same time, the cells–microcarriers were immobilized after culturing for 24 h, 72 h, and 120 h with 0.25% glutaraldehyde solution and then dehydrated gradually by ethanol solution, followed by the SEM to investigate cell adhesion and growth. In addition, the process of cell adhesion and the subsequent spreading and proliferation on the surface of microcarriers was observed with an inverted phase contrast microscope after culturing for 30 min, 24 h, and 72 h. Further, trypsin digestion was used to study the process of cell desorption from the three kind microcarriers after 5 days culture. Besides, the detached cells were harvested and reseeded into 24-well plates whose viability was tested by live/dead staining after culturing for 24 h and 168 h.

### 2.8. Cell Adhesion, Proliferation, and Differentiation on Microcarriers

One hundred microliters of cell suspension was seeded on microcarriers in a 96-well plate at a cell density of 5 × 10^4^ cells/well, while the cell suspension without microcarriers was set as the control group. To determine cell adhesion efficiency, in brief, supernatant and microcarriers were removed at 10 min, 20 min, and 30 min post seeding, followed by addition of 100 μL complete medium and 10 μL/well CCK-8 reagent, and incubation at 37 °C for 3 h. Then, the mixtures of culture medium and CCK-8 (100 μL) were used to measure optical density (OD) at the emission wavelengths of 562 nm and 620 nm via an Enzyme-linked immune detector. The cell adhesion rate was then calculated by comparing the difference in OD between the control group and the experimental group. Cell proliferation rate was further measured at 24 h, 72 h, and 120 h post cell seeding using the same method.

The MC3T3-E1 cells were inoculated directly onto the three kinds of microcarriers, and Alkaline Phosphatase (ALP) staining was performed after one week of osteogenic induction. Equally, MC3T3-E1 cells (1 × 10^5^ cells/per well) were inoculated into the 24-well plate and cultured for 24 h prior to the addition of microcarriers, followed by ALP staining as above. Also, the three kinds of microcarriers with the same weights were respectively placed in a low adhesion 6-well plate, followed by the seeding of 100 μL of cell suspension with a density of 5 × 10^6^ cells/mL. After culturing for 7 and 14 days, ALP activity of MC3T3-E1 cells was assayed using an alkaline phosphatase assay kit, and the result was normalized to the total protein content.

### 2.9. Statistical Analysis

All experimental results were expressed as mean ± SD (Standard Deviation). The statistical significance of differences within each group was evaluated by one-way ANOVA followed by the *t*-test analysis using OriginPro (v7.5, OriginLab Corporation, Northampton, MA, USA). It was regarded as a significant difference when the value of *p* < 0.05.

## 3. Results and Discussion

### 3.1. The Particle Size Distribution of Microcarriers

**Figure 1:**
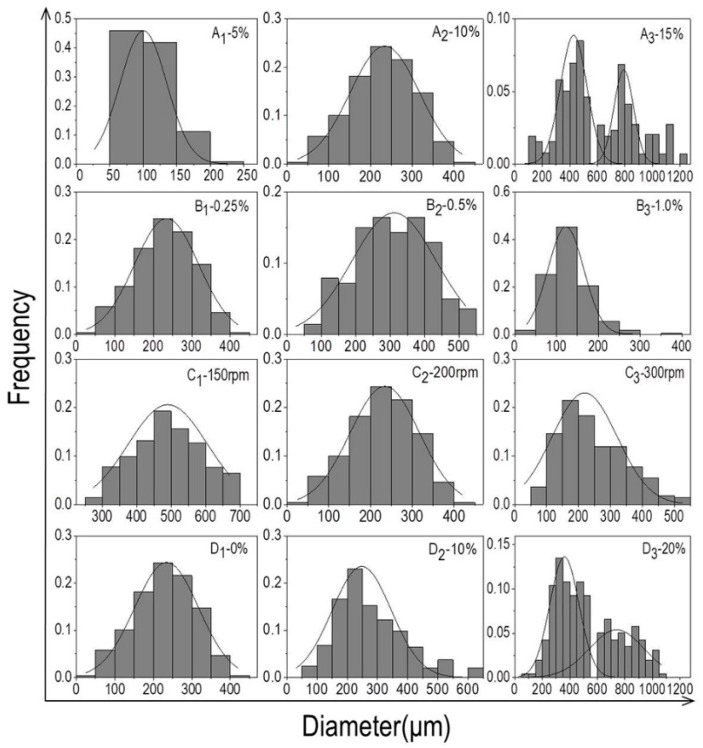
(**A_1_**–**A_3_**) Histograms of particle size distribution of PLLA microcarriers prepared with different concentrations of PLLA (**A_1_**–**A_3_**: 5%, 10%, 15%; the gelatin concentration was fixed at 0.25% and the stirring speed at 200 rpm); (**B_1_**–**B_3_**) histograms of the particle size distribution of PLLA microcarriers prepared by different concentrations of gelatin (**B_1_**–**B_3_**: 0.25%, 0.5%, 1%; the PLLA concentration was kept constant at 10% and stirring speed at 200 rpm); (**C_1_**–**C_3_**) histograms of particle size distribution of PLLA microcarriers prepared by different stirring speeds (**C_1_**–**C_3_**): 150 rpm, 200 rpm, 300 rpm; the concentration of PLLA and gelation was held unchanged at 10% and 0.25%, respectively); (**D_1_**–**D_3_**) histograms of particle size distribution of PLLA/nHA microcarriers prepared by different concentrations of nHA (**D_1_**–**D_3_**): 0%, 10%, 20%; the concentration of PLLA and gelation was kept unchanged at 10% and 0.25%, and stirring speed at 200 rpm).

The average particle size of the microcarriers increased from 109.1 μm to 544.9 μm when the concentration of PLLA grew from 5% to 15% with the gelatin concentration at 0.25% and the stirring speed at 200 rpm (Figure 1A_1_–A_3_). The reason was the oil phase became more viscous with the increasing concentration of PLLA, resulting in the formation of larger oil droplets through emulsion dispersion, thus the particle size of the microcarriers increased. This was consistent with some relevant studies [36,37]. For example, Cheng et al. [36] have examined the impact of phase viscosity on droplet size by using different octadecane-to-corn oil ratios in the oil phase and different glycerol-to-water ratios in the aqueous phase. Results showed that the mean droplet diameter plot increased with increasing viscosity ratio of the oil phase. McClements et al. [37] interpreted some potential mechanisms about the influence of oil phase viscosity on droplet size: increased droplet fragmentation due to increased disruptive shear stresses; decreased droplet re-coalescence due to decreased droplet collision frequency; increased droplet re-coalescence due to a reduction in emulsifier adsorption rate. When the PLLA concentration was fixed at 10% and the stirring speed at 200 rpm, the size drop from 316.0 μm to 129.2 μm was attributed to the increased concentration of gelatin from 0.25% to 1% (Figure 1B_1_–B_3_). In general, if the solubility of PLLA remained unchanged, the viscosity and dispersion of the aqueous phase would increase with extra element of gelatin, therefore, the oil phase can be dispersed into small oil beads in a short time, and the resistance of the microcarriers to collide with each other magnified so that the probability of collision between each other was lowered. As a result, the particle size decreased with the fall of surface energy. Based on this principle, the surface energy of the oil phase drops can be further reduced by adding gelatin solution, resulting in a size drop. As shown in the diagram, increased stirring speed from 150 rpm to 300 rpm with fixed PLLA concentration at 10% and gelatin concentration at 0.25% (Figure 1C_1_–C_3_), the particle size of microcarriers significantly reduced from 521 μm to 102 μm. The explanation for this finding was that the accelerated stirring speed gradually increased the shearing force of the solution, and hence the oil droplets were dispersed into a smaller size. With the addition of nHA content, at first, the diameter of microcarriers decreased slightly and later increased with the unceasing addition of nHA (Figure 1D_1_–D_3_). nHA, as a hydrophilic compound, tended to cover the surface of the oil phase when added, and constantly attracted the water molecules to reduce the surface energy of the oil phase. Simultaneously, nHA can also increase the viscosity of the oil phase, resulting in the formation of larger oil droplets in the process of emulsification. Based on the above analysis, the following experimental conditions were selected for subsequent cell-microcarriers study: 10% PLLA, 0.25% gelatin, and 20% nHA with rotational speed of 200 rpm. The preparation of particles with smaller dimensions will continue to be explored in future experiments and be used for three-dimensional cell culture.

### 3.2. Morphology, Component Analysis, Protein Adsorption, and Degradation of Microcarriers

**Figure 2:**
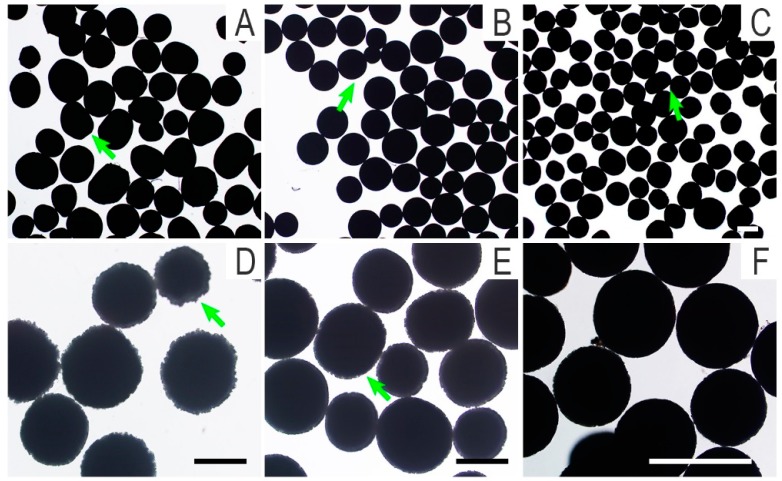
Outline drawings of microcarriers (**A**): PLLA microcarriers; (**B**): PLLA/nHA microcarriers; (**C**,**F**): PLLA/nHA/Ch microcarriers; (**D**): the PLLA/nHA microcarriers treated with sodium hydroxide for 20 min; (**E**): the PLLA/nHA microcarriers treated with sodium hydroxide for 10 min. (**A**–**C**, **D**,**E**, **F**: 40×, 100×, 200×; Scale: 250 μm).

The outlines of PLLA, PLLA/nHA, and PLLA/nHA/Ch microcarriers observed by inverted phase contrast microscopy are shown in Figure 2A–C. Macroscopically, the degree of sphericity of PLLA/nHA microcarriers was superior compared to the PLLA and PLLA/nHA/Ch microcarriers. The surface morphology of PLLA/nHA microcarriers with the alkaline treatment of 20 min and 10 min (Figure 2D,E) indicated that the surface erosion of microcarriers was affected by the time of alkaline treatment, also resulting in the formation of a serrated surface profile. The dissolution of microcarriers was aggravated with the extension of time. For this reason, the time of alkaline treatment was set to 10 min in the subsequent experiments. The microcarriers after crosslinking chitosan (Figure 2F) still kept the zigzag profile, which was not significantly different from that of alkaline-treated microcarriers. These microcarriers were screened in advance to remove the unformed impurities. Their average diameter was obtained by calculating the diameters of 100 particles (Table 1). It was found that the average diameter of PLLA microcarriers was 291.9 ± 30.7 μm, and the average diameter of PLLA/nHA microcarriers was 275.7 ± 30.6 μm when doped with a certain amount of nHA, while the average diameter of PLLA/nHA/Ch microcarriers turned to be 269.4 ± 26.3 μm. These phenomena explained that the addition of hydroxyapatite improved the sphericity and reduced the average diameter of PLLA microcarriers to a certain extent, and the average diameter of PLLA/nHA/Ch microcarriers was further decreased and their surface roughness was increased by alkaline treatment.

**Figure 3:**
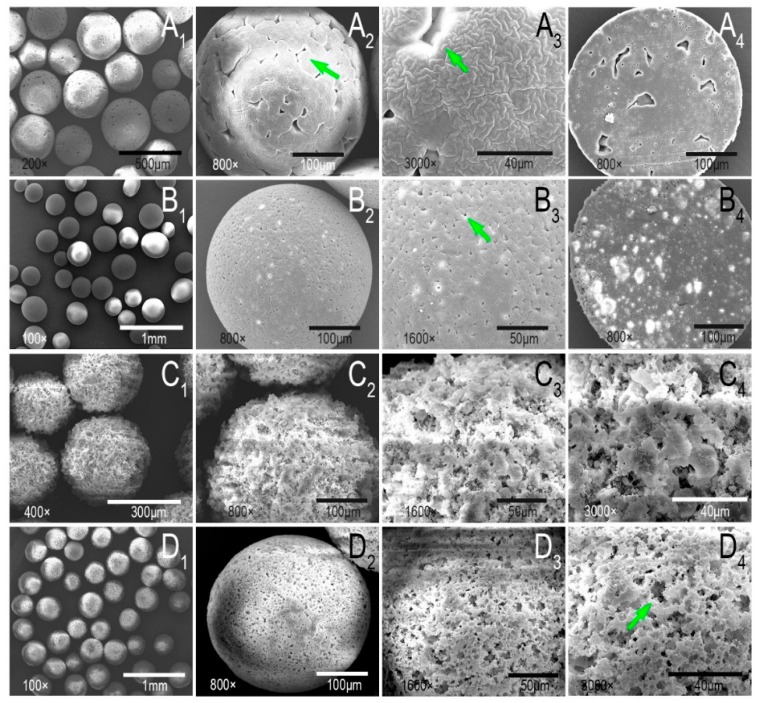
(**A_1_**–**A_3_**): The surface morphology of PLLA microcarriers; (**B_1_**–**B_3_**): The surface morphology of PLLA/nHA microcarriers; (**A_4_**,**B_4_**): The cross-sectional morphology of PLLA and PLLA/nHA microcarriers; (**C_1_**–**C_4_**): The surface morphology of PLLA/nHA microcarriers after alkaline treatment of 10 min; (**D_1_**–**D_4_**): The surface morphology of PLLA/nHA/Ch microcarriers coated with chitosan.

SEM observation of PLLA and PLLA/nHA microcarriers (Figure 3A,B) indicated that the presence of nHA changed the surface morphology of PLLA microcarriers resulting in a porous surface. The mechanism of emulsion solvent evaporation has been investigated by Rosca et al. [38]. It was concluded in their research that emulsion solvent evaporation (ESE) was mainly a two-step process: the emulsification of a polymer solution containing the encapsulated substance, followed by particle hardening through solvent evaporation and polymer precipitation according to the optical microscopic observations corroborated with laser diffractometry analysis. The second step, with the solvent transporting out from the emulsion droplets, determined the particle morphology. In addition, Hong et al. [39] thought the possible reason for the morphology of the PLLA microcarriers in this manuscript was that the shear stress might create defects during the solvent evaporation process. Also, small pores appeared on the surface of the microcarriers when they mixed the n-hexane with PLLA/dichloromethane solution. They believed that it was caused by the removal of the non-solvent. Therefore, in this study, the morphological differences between PLLA and PLLA/nHA microcarriers should be attributed to the hydrophilicity of nHA and its effect on the volatilization of dichloromethane, thereby leading to the formation of many uniformly distributed small and closed pores. The internal structure of PLLA/nHA microcarriers was more compact with prominent white luminous spots of agglomerated nHA (Figure 3B_4_). The topography of alkaline-treated microcarriers (10 min) and chitosan cross-linking in Figure 3C,D showed that the surface of PLLA/nHA/Ch microcarriers became uneven, and the closed pores formed by solvent evaporation were interconnected, which increased the specific area of the microcarriers and enhanced the surface roughness.

**Figure 4:**
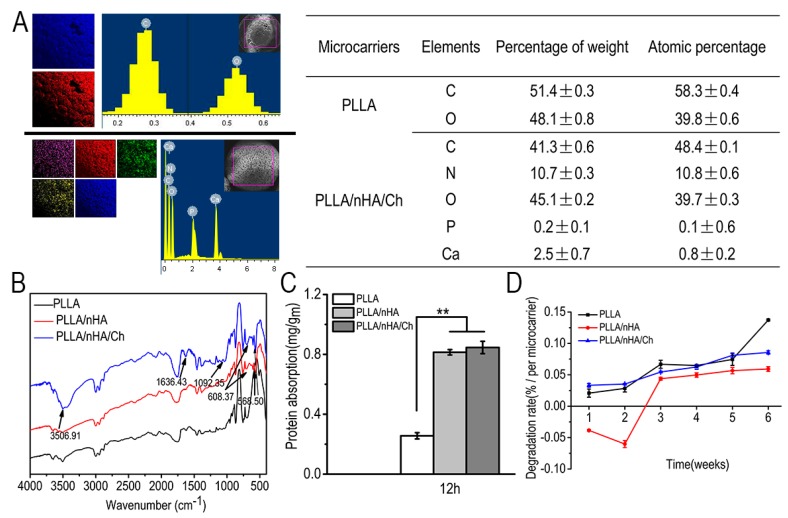
(**A**): Semi-quantitative analysis of surface elements of PLLA microcarriers (including carbon and oxygen) and PLLA/nHA/Ch microcarriers (including carbon, nitrogen, calcium, phosphorus, and oxygen); (**B**): Analyses of characteristic chemical functional groups of the three kinds of microcarriers by Fourier transform infrared spectroscopy; (**C**): Amount of protein adsorbed on the surfaces of microcarriers after incubation for 12 h at 37 °C. Data were expressed as mean ± SD (*n* = 6, ** *p* < 0.01); (**D**): The degradation of the three kinds of microcarriers in PBS solution.

The EDS spectrometer provided a semi-quantitative elemental analysis of the specific area of a sample whose accuracy was from 1% to 5% and depth from 1 μm to 5 μm. It cannot guarantee that the same square box for PLLA and PLLA/nHA/Ch samples was drawn. Hence, each sample was determined in triplicate, and only one set of the three tests was displayed in Figure 4A. Meanwhile, as shown in the table of Figure 4A, the mean ± SD values of the specific elements of the intercepted region of microcarriers, which was calculated based on the triplicate tests of each sample, illustrated that the addition of nHA and crosslinking of chitosan had been successfully realized because calcium, phosphorus, and nitrogen were detected in the PLLA/nHA/Ch microcarriers, but not in the PLLA microcarriers. A quantitative analysis of nitrogen is listed in Table 2, which further stated that the presence of chitosan as nitrogen was not detected in either PLLA or PLLA/nHA microcarriers. The Fourier transform infrared spectrum of the characteristic chemical groups of microcarriers is shown in Figure 4B. At 568 cm^−1^ and 608 cm^−1^, the characteristic absorption peaks of phosphate groups appeared, indicating that doping with nHA was achieved. Meanwhile, the enhanced C=O peak (acrylamide band I) and the absorption peak of acrylamide band II and III at 1636 cm^−1^ and 1092 cm^−1^ also demonstrated that chitosan was successfully coated on the surface of microcarriers. Protein adsorption of materials was influenced by factors such as surface charge, roughness, or the hydrophilicity of the material, which affected cell interaction with materials. In Figure 4C, the amount of protein adsorption of the microcarriers increased when nHA was added, owing to the calcium ions in nHA which can bind to negatively charged protein. Although the surface roughness of PLLA/nHA/Ch microcarriers was the largest, the protein adsorption of the PLLA/nHA/Ch microcarriers had no obvious significance with that of PLLA/nHA microcarriers. This was mainly due to the partial loss of nHA caused by the dissolution of microcarriers in the alkaline solvent. Some studies have investigated the correlation between nHA and protein adsorption. For example, Kandori et al. [40,41] stated that calcium atoms would be exposed on the Hap surface by dissolution of OH ions at the particle surface to produce calcium ions or positively charged sites to bind to acidic groups of proteins after dispersing nHA particles in aqueous media. In Figure 4D, it was found that the degradation of PLLA microcarriers increased with time, and it was slowed down by the addition of hydroxyapatite. The decreased degradation ratio of PLLA/nHA in the 2-week period was believed to be caused by the mineralization of nHA in the PBS buffer solution. Kanjwal et al. [42] studied the mineralization of nanohydroxyapatite/PCL in SBF, a simulated body fluid. Its composition was somewhat similar to PBS, including NaCl, KCl, K_2_HPO_4_. The principle of mineralization was that the incubation of the introduced PCL/HAp in the SBF led to the formation of excessive apatite due to stimulating the crystallization of the biological apatite. However, with the extension of time, the degradation of PLLA eventually dominated, resulting in the decline of the weight of microcarriers. But for PLLA/nHA/Ch microcarriers this condition did not happen. This was mainly because of the alkaline treatment of the PLLA/nHA/Ch microcarriers, which resulted in the loss of part of nHA. Meanwhile, although chitosan also had the advantage of retarding the degradation of PLLA/nHA/Ch microcarriers, the surface erosion caused by alkaline treatment promoted the degradation to a certain extent [43]. As Lyu et al. [44] pointed out, the degradation mechanism of PLLA can be either surface or bulk erosion depending on the competition between the diffusion of water molecules into PLLA and the rate of the hydrolysis reactions. It was also confirmed by elemental analysis that chitosan was successfully crosslinked with microcarriers (Table 2).

### 3.3. The Morphology and Osteogenic Differentiation of MC3T3-E1 Cells

**Figure 5:**
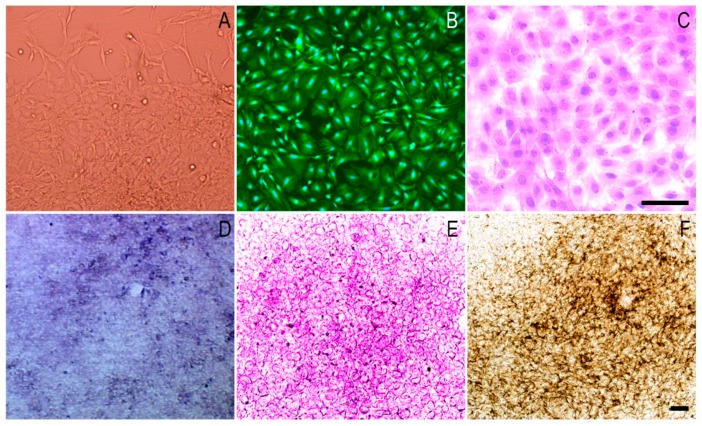
Phase contrast microscopy images of the morphology and differentiation of MC3T3-E1 cells. (**A**): cell morphology of MC3T3-E1cells; (**B**): Calcein-AM staining of the seventh generation of cells; (**C**): Hematoxylin-eosin (HE) staining of the seventh generation of cells; (**D**): Alkaline phosphatase staining after one week of osteogenic induction; (**E**): Alizarin red S staining after three weeks of osteogenic induction; (**F**): Von-Kossa staining after four weeks of osteogenic induction. Scale: 250 μm.

Figure 5A–C shows the adhesion and spreading pattern of MC3T3-E1 cells by light microscopy, Calcein-AM staining, and HE staining. After one week of osteogenic induction, alkaline phosphatase staining revealed that the cells had high expression of alkaline phosphatase, characteristic of the first stage of differentiation (Figure 5D). Also, red calcium nodules were noted by Alizarin Red S staining (Figure 5E) and a brown-black substance was observed with Von-Kossa staining (Figure 5F). It was suggested from the above tests that MC3T3-E1 cells with good proliferation and high osteogenic differentiation ability in vitro could be used for subsequent cell–microcarrier complex cultivation.

### 3.4. Live/Dead Staining of Cells on Microcarriers

**Figure 6:**
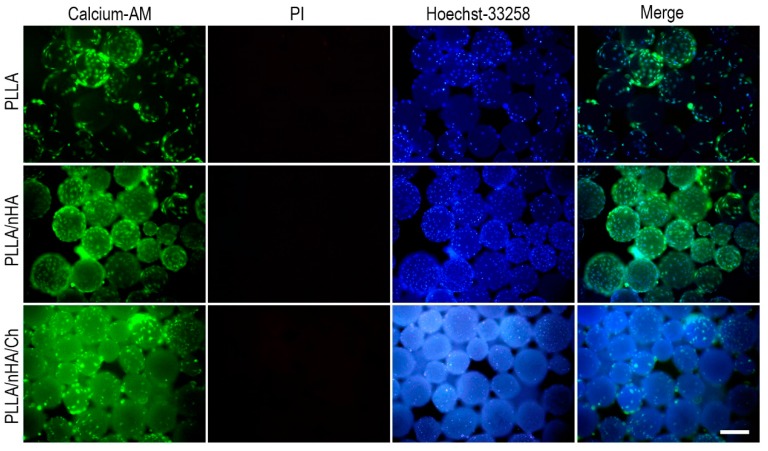
Live/dead staining of MC3T3-E1 cells of P7 passage cultured for 24 h on the surface of the three microcarriers, which were respectively the Calcein-AM, PI and Hoechst33258 staining, and their overlay composite graph. Scale: 250 μm.

**Figure 7:**
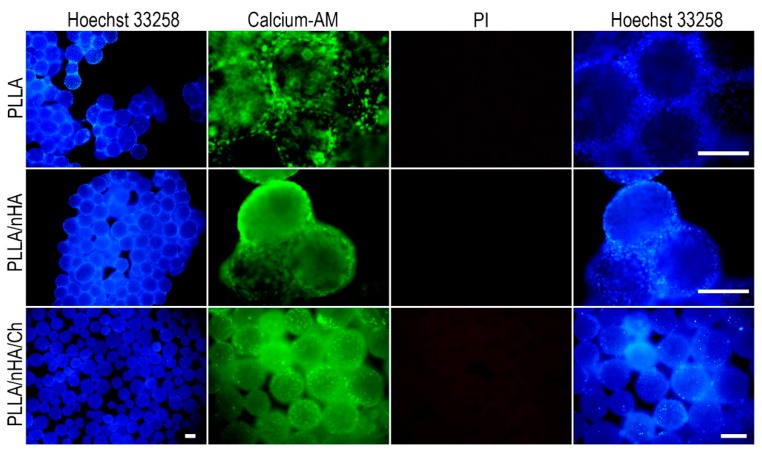
Live/dead staining of MC3T3-E1 cells of P7 passage cultured for 120 h on the surface of the three microcarriers. Scale: 250 μm.

The live/dead staining of cells cultured on the surface of microcarriers conducted at 24 h (Figure 6) showed that MC3T3-E1 cells had adhered and spread on the surface of microcarriers and maintained high cell viability, especially on the PLLA/nHA microcarriers. The observation of cells on PLLA/nHA/Ch microcarriers was difficult because the microcarriers were also stained due to the surface-modified chitosan. After 120 h (Figure 7), it was found that originally dispersed PLLA and PLLA/nHA microcarriers were packaged by a large number of cells. However, this did not appear on the PLLA/nHA/Ch microcarriers, which was speculated to be due to the fact that the rough surface of the PLLA/nHA/Ch microcarriers did not significantly promote cell proliferation at the same culturing time compared with the PLLA/nHA microcarriers.

### 3.5. Cell Adhesion, Detachment, and Differentiation on Microcarriers

**Figure 8:**
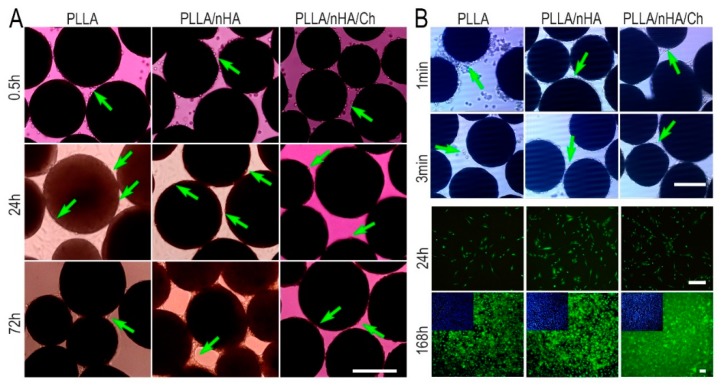
(**A**) The time-course of adhesion and spread of MC3T3-E1 cells of P7 passage on the surface of the three microcarriers with the inoculation density of 1 × 10^5^ cells/mL containing the stages of contact, adhesion, spreading, and proliferation; (**B**) The detachment process of MC3T3-E1 from microcarriers and live/dead staining of the detached cells which were reseeded into 24-well plates and cultured for one week. Scale: 250 μm.

The process of cell adhesion and spreading on the microcarriers was exhibited in Figure 8A. In the first 30 min, the cells initially came into contact with the surface of the three kinds of microcarriers in the shape of sphere, and then gradually became ellipsoid, flattened, and stretched out to adhere to the surface of microcarriers within 24 h. After 120 h, cells had proliferated in large numbers and encapsulated the entire surface of the microparticles. However, this was not found on the PLLA/nHA/Ch microcarriers, implying that they did not produce an active effect on the expansion of MC3T3-E1 cells. After cultured for 7 days, the desorption process of MC3T3-E1 cells in three minutes under the action of trypsin (Figure 8B) showed easy cell detachment from PLLA microcarriers, but not from PLLA/nHA/Ch microcarriers in the same time interval, indicating that the adhesion between MC3T3-E1 cells and PLLA/nHA/Ch microcarriers was the strongest, and that of PLLA microcarriers was the weakest. The cells which were reseeded into the 24-well plate were found to have a good proliferation state after a week of culture, and show an obvious difference with the morphology of cells in 2D (two-dimensional) culture (Figure 5A,B), illustrating that the cells detaching from the three kinds of microcarriers retained good adhesion and division capability.

**Figure 9:**
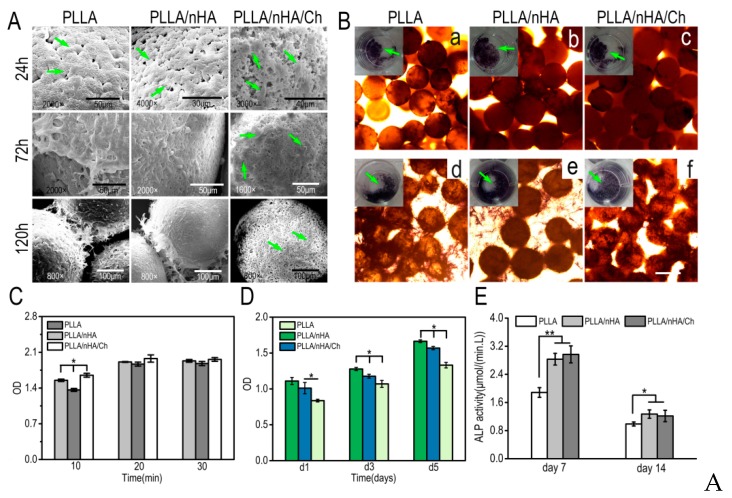
(**A**): Adhesion, spread, and proliferation of MC3T3-E1 cells cultured on the surface of microcarriers for 24 h, 72 h, and 120 h observed by scanning electron microscopy; (**B**): a–c: Alkaline phosphatase staining of MC3T3-E1 cells inoculated onto the surface of microcarriers after one-week osteogenic induction (the green arrows indicated the cells-microcarriers complex in the 24-well plate stained by alkaline phosphatase). d–f: Alkaline phosphatase staining of MC3T3-E1 cells firstly inoculated into the 24-well plate and cultured for 3 days, then adhered to the microcarriers. Scale: 250 μm; (**C**): The adhesion determination of MC3T3-E1 cells on the surface of microcarriers; (**D**): The proliferation detection of MC3T3-E1 cells on the surface of microcarriers (*n* = 3, * *P* < 0.05); (**E**): ALP activity assay of MC3T3-E1 cells on the three kinds of microcarriers (*n* = 3, * *p* < 0.05, ** *p* < 0.01).

The spread morphology of cells on the surface of microcarriers observed by SEM (Figure 9A) showed that the proliferation of cells on the first two kinds of microcarriers was significantly better than on PLLA/nHA/Ch microcarriers after culturing for 72 h. The cells were seeded on the three kind microcarriers by two methods, as mentioned above. In Figure 9B, the green arrows indicate the cell–microcarrier complexes in the 24-well plate stained by alkaline phosphatase after cultivation for seven days in osteogenic medium. It can be seen all of them performed an obvious expression with a dark blue-violet color which was similar to Figure 5D. It was also found that the cells growing in the 24-well plate would migrate to the microcarriers, because many cells had appeared on the top surface of the microcarriers, suggesting that these three kinds of microcarriers prepared using gelatin as dispersant had a certain cellular affinity. As shown in Figure 9C, the cell adhesion rate was found to be significantly different on these three kinds of microcarriers in the first 10 min post cell seeding, followed by a slight increase until they reached the stable condition. The highest cell adhesion efficiency was found in the group of PLLA/nHA/Ch. This could be explained by the increased surface roughness, which was conducive to the retention of cells. The cell proliferation rate in Figure 9D demonstrated that the PLLA/nHA microcarriers had the optimum proliferation-promoting characteristics, whereas the cross-linked modified microcarriers were not favorable. As it was found by the front observation of SEM, although the modification of the microcarriers increased the surface roughness and created interconnected microchannels, these microchannels were too small to help the growth of MC3T3-E1 cells, with evidence that all cells spread out flat on the surface. The quantitative detection of ALP (Figure 9E) showed that the cells on the PLLA/nHA/Ch microcarriers had the highest ALP activity. Although the cells grew slowly on the surface of PLLA/nHA/Ch microcarriers, the cells could still fully coat the microcarriers after a certain period of culturing. Some studies have found that surface roughness plays an extremely subtle role in cell proliferation and differentiation, and only in a suitable roughness range can the best effect be achieved. For example, Borsari et al. [45] concluded in their study that values of roughness that overcome specific limits were not correctly seen by cells and HA coating had a synergic positive effect on cells only when the proper roughness is present. Zan et al. [46] made β-TCP/chitosan composite microspheres with four kinds of surface roughness and it was found that the optimum roughness of the surface was 2.0 μm. Hu et al. [47] have found that a combination of low surface roughness and high stiffness of the substrate appeared to be the most favorable for proliferation and myogenic-differentiation of C2C12 cells, while in contrast, hMSCs demonstrated a preference for higher surface roughness and stronger micro/nano-scale surface patterns. Zhang et al. [48] demonstrated that a rough stainless steel surface improved cell adhesion and morphology, while HA coating which had a higher roughness contributed to superior cell adhesion, but inhibited cell proliferation. In a word, a rough surface has both its pros and cons on cell adhesion and proliferation.

Currently, many researchers focus on combinations of synthetic polymer materials, natural polymer materials, and bioactive ceramics to prepare composite materials for tissue engineering and regeneration [49,50,51], not only making up for hydrophobicity and the lack of adhesive ligands of synthetic polymers, but also resolving the readily degradable and structurally unstable disadvantages of natural polymers, and most importantly, enhancing the proliferation and differentiation of cells. In this study, gelatin with high biocompatibility was utilized as the dispersant, different from other dispersants such as polyvinyl alcohol, toluene, and Tween, which made the preparation process safer and more concise. Even if the dispersant remained on the surface of the microcarriers, it would not cause toxicity to the cells. Considering the hydrophobicity of PLLA, chitosan was further coated on the surface of the microcarriers after finishing the construction of PLLA/nHA composite microcarriers. It was found that the PLLA/nHA microcarriers had the greatest proliferation-promoting effect, while the PLLA/nHA/Ch microcarriers had the strongest attachment with MC3T3-E1 cells. The cells on the PLLA/nHA/Ch microcarriers exhibited optimal osteogenic expression. Generally, the function of PLLA microcarriers on osteoblast-like cell cultivation has been greatly enhanced by the doping of nanohydroxyapatite and further modification with chitosan.

## 4. Conclusions

In this study, three kinds of microcarriers were prepared based on physical doping and surface modification, namely Poly (l-lactic) acid (PLLA), PLLA/nanohydroxyapatite (PLLA/nHA), and PLLA/nHA/Chitosan (PLLA/nHA/Ch) using gelatin as the dispersant. The physicochemical properties of the three kinds of microcarriers and their functional performance in MC3T3-E1 cell culture were compared. The surface roughness and protein adsorption of the microcarriers were enhanced with the doping of nHA and coating of chitosan. Murine MC3T3-E1 cells with demonstrated osteogenic differentiation activity were seeded onto the surface of microcarriers to investigate the cytocompatibility, and the results showed that MC3T3-E1 cells could adhere and proliferate on the three kinds of microcarriers, and the PLLA/nHA microcarriers had the greatest proliferation-promoting effect, while the PLLA/nHA/Ch microcarriers exhibited the strongest attachment with MC3T3-E1 cells. Besides, the cells on the PLLA/nHA/Ch microcarriers exhibited optimal osteogenic expression. Generally, chitosan improved the microcarrier characteristics of cell adhesion and differentiation, and nanohydroxyapatite improved their sphericity and promotion of cell proliferation. In conclusion, the modified microcarriers provide a promising strategy for 3D cultivation of osteoblast-like cells, and might be good candidates for bone tissue engineering. Future work will study the impact of relevant experimental parameters, such as alkaline treatment time and concentration of chitosan, on the biocompatibility and morphology of PLLA/nHA/Ch microcarriers. Three-dimensional dynamic experiments utilizing the modified PLLA microcarriers will also be carried out to seek the appropriate ex vivo environment for cell survival and propagation.

## Figures and Tables

**Table 1:** Analysis of mean diameter and sphericity of microcarriers.

Microcarriers	PLLA	PLLA/nHA	PLLA/nHA/Ch
Mean diameter/μm	291.9 ± 30.7	275.7 ± 30.6	269.4 ± 26.3
Sphericity	++	+++	+

Note: the sphericity of microcarriers was artificially judged by three viewers who were not involved in this experiment. The three plus signs indicated that the sphericity of the microcarriers was the best.

**Table 2:** Nitrogen content of microcarriers measured by an elemental analyzer.

Microcarriers	Elemental Fraction (wt %)		
	C	N	H
PLLA	41.93 ± 0.11	0	4.80 ± 0.03
PLLA/nHA/Ch	46.96 ± 0.13	0.03 ± 0.01	5.22 ± 0.01
